# Automated Non-Coplanar VMAT for Dose Escalation in Recurrent Head and Neck Cancer Patients

**DOI:** 10.3390/cancers13081910

**Published:** 2021-04-15

**Authors:** Kaley Woods, Robert K. Chin, Kiri A. Cook, Ke Sheng, Amar U. Kishan, John V. Hegde, Stephen Tenn, Michael L. Steinberg, Minsong Cao

**Affiliations:** 1 Department of Radiation Oncology, University of California, Los Angeles, CA 90095, USA; KaleyWoods@mednet.UCLA.edu (K.W.); RKChin@mednet.ucla.edu (R.K.C.); ksheng@mednet.ucla.edu (K.S.); aukishan@mednet.ucla.edu (A.U.K.); JHegde@mednet.ucla.edu (J.V.H.); stenn@mednet.ucla.edu (S.T.); MSteinberg@mednet.ucla.edu (M.L.S.); 2 Department of Radiation Oncology, Oregon Health & Science University, Portland, OR 97239, USA; cooki@ohsu.edu

**Keywords:** HyperArc, SBRT, reirradiation, recurrent head and neck cancer, non-coplanar VMAT

## Abstract

**Simple Summary:**

The ability to escalate the radiation dose to head and neck tumors has been shown to offer improved local control, and consequently, survival for recurrent head and neck cancer (rHNC) patients. This study evaluates the HyperArc automated non-coplanar planning technique (originally developed for intracranial treatment) for 20 rHNC patients, and compares this technique to conventional planning methods. HyperArc enables significant tumor dose escalation, with average increases in mean target dose of over 11.5 Gy (26%), while maintaining clinically-equivalent doses to nearby organs. Our results show that the average probability of tumor control is 23% higher for HyperArc than conventional techniques.

**Abstract:**

This study evaluates the potential for tumor dose escalation in recurrent head and neck cancer (rHNC) patients with automated non-coplanar volumetric modulated arc therapy (VMAT) stereotactic body radiation therapy (SBRT) planning (HyperArc). Twenty rHNC patients are planned with conventional VMAT SBRT to 40 Gy while minimizing organ-at-risk (OAR) doses. They are then re-planned with the HyperArc technique to match these minimal OAR doses while escalating the target dose as high as possible. Then, we compare the dosimetry, tumor control probability (TCP), and normal tissue complication probability (NTCP) for the two plan types. Our results show that the HyperArc technique significantly increases the mean planning target volume (PTV) and gross tumor volume (GTV) doses by 10.8 ± 4.4 Gy (25%) and 11.5 ± 5.1 Gy (26%) on average, respectively. There are no clinically significant differences in OAR doses, with maximum dose differences of <2 Gy on average. The average TCP is 23% (± 21%) higher for HyperArc than conventional plans, with no significant differences in NTCP for the brainstem, cord, mandible, or larynx. HyperArc can achieve significant tumor dose escalation while maintaining minimal OAR doses in the head and neck—potentially enabling improved local control for rHNC SBRT patients without increased risk of treatment-related toxicities.

## 1. Introduction

Between 15 and 50% of head and neck cancer patients treated with radiation experience locoregional recurrence, which is the most common cause of failure [1,2]. Although salvage surgery is the preferred treatment option in the case of recurrence, achieving 5-year overall survival rates of up to 40% [3], many patients are not surgical candidates due to extensive tumor involvement or poor overall health [4]. Radiation therapy with or without adjuvant chemotherapy is the next best option, but reirradiation poses high risks of severe toxicity, including fistula, ulceration, and carotid blowout syndrome [5,6,7]. Despite these risks, the 2-year overall survival rates following conventionally-fractionated reirradiation are only 15–25%. The delivery of higher fractional doses with stereotactic body radiation therapy (SBRT) has been shown to significantly improve local control, with reported 2-year overall survival rates as high as 50% [7,8]. However, even with SBRT, severe toxicity rates can be over 25% and fatal carotid artery blowout rates as high as 17% when full prescribed reirradiation doses are delivered [7]. Therefore, the ability to escalate target doses while sparing normal tissue could improve local control, and consequently survival for rHNC patients.

The potential for non-coplanar beam angles to increase dose conformity has long since been established [9]. However, manual non-coplanar beam selection, particularly for extracranial treatment, is unintuitive, inefficient, and increases the probability of patient-machine collision. One innovative technique for safely delivering optimized, highly non-coplanar IMRT with a conventional C-arm linac is 4π radiotherapy [10]. The 4π framework first uses a computer-aided design model to identify a patient-specific set of deliverable beams, eliminating any beam angles that could potentially cause collision between the gantry and the patient or couch. Integrated beam orientation and fluence map optimization are then performed to create highly non-coplanar, conformal treatment plans. 4π radiotherapy has been shown to enable significantly more conformal dose distributions than conventional planning techniques in a wide variety of treatment sites, including the liver [10,11], lung [12], prostate [13,14], brain [15,16], and head and neck [8,17]. A 4π VMAT method has also been developed with dynamic gantry and couch rotation, with dosimetric benefits demonstrated for brain, lung, and prostate cancer [18]. Rwigema et al. showed that the incorporation of non-coplanar beams in head and neck SBRT with the 4π radiotherapy technique enabled substantial reductions in OAR doses, including mean dose reductions of >50% to the spinal cord, brainstem, parotids, and larynx, as well as significant reductions in the 50% dose spillage volume and the probability of late toxicities [8]. In addition to these dosimetric advantages, the safety and feasibility of 4π treatment delivery were demonstrated in a prospective clinical trial for recurrent high grade glioma patients [19].

Although 4π radiotherapy could, therefore, be a promising solution for safely escalating target doses in the rHNC population, this optimization algorithm is not clinically available. The automated delivery of non-coplanar beams with 4π has also yet to be approved by the FDA, which heavily limits its clinical applications. A commercial tool (HyperArc, Varian Medical Systems, Palo Alto, CA, USA) was recently developed in response to 4π radiotherapy and has made automated non-coplanar VMAT planning and delivery clinically feasible. HyperArc was developed for intracranial treatment, and its benefits for brain stereotactic radiosurgery have been widely demonstrated [20,21,22,23,24]. However, this technique can also be adapted for many head and neck cases with a modified planning strategy and workflow. This novel use of HyperArc planning and delivery has not yet been fully explored.

In this study, we explored the use of the automated non-coplanar VMAT planning technique for a wide range of sites in the head and neck and evaluated its ability to escalate target doses for improved tumor control probabilities in rHNC patients.

## 2. Materials and Methods

### 2.1. Patient Selection

This study included twenty rHNC patients. Institutional Review Board (IRB) review and approval (ID 18-001247) was obtained for this study, and all the patients in the clinical trial were given their informed consent for inclusion before they participated in the study. Eleven patients had been reirradiated with 5 fraction SBRT (typically to 40 Gy) and were retrospectively re-planned using the HyperArc technique. Nine patients are part of an ongoing clinical trial at our institution for which both HyperArc and conventional VMAT plans (referred to hereafter as conventional plans) were created prior to treatment. The patient characteristics and details for these conventional plans are given in Table 1. These patients had a range of treatment sites in the head and neck, including the sinus, oral cavity, hypopharynx, neck, and supraclavicular nodes, the majority of which were squamous cell carcinoma. The gross tumor volume (GTV) was delineated by the physician using images from the CT simulation and the previously obtained PET/CT and MRI, and a 2 mm expansion was added for treatment setup error to comprise the planning target volume (PTV). The PTV volumes ranged from 4.9 to 80.6 cm^3^ with an average of 35.2 cm^3^.

**Figure 1:**
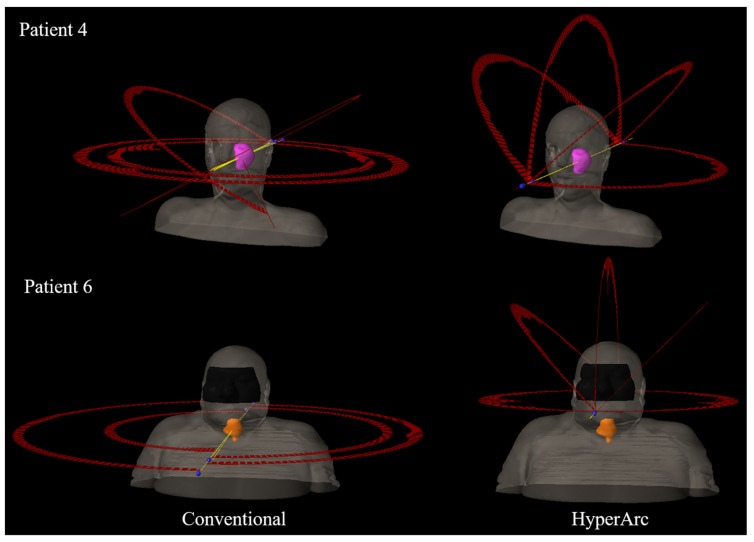
Beam arrangements for the conventional (**left**) and HyperArc (**right**) plans for two rHNC patients in this study. The PTV structures are shown in pink (Patient 4) and orange (Patient 6), and the yellow lines indicate the central axes for each beam.

A dose of 40 Gy was prescribed to 95% of the PTV, except in a few cases where coverage was sacrificed to protect adjacent OARs. Since these patients all received prior radiation, and especially since the dose distributions for many of these prior treatments could not be obtained, the priority was to spare the dose to critical organs as much as possible. Highly inhomogeneous target doses were allowed, and hotspots in the center of the GTV were encouraged to increase the mean target dose. The plans were created with the Eclipse treatment planning system (version 15.6, Varian Medical Systems, Palo Alto, CA, USA) using a Varian High Definition 120 MLC, energy of 6X-FFF, and dose rate of 1400 MU/min. All conventional plans used RapidArc VMAT with up to two full or five partial arcs, and only four plans utilized non-coplanar beam angles. Examples of two conventional plan beam arrangements, with and without non-coplanar beams, are shown in Figure 1. All planning was performed or reviewed by an experienced planner with over 15 years of SBRT planning experience to ensure consistency in the planning strategy and quality.

### 2.2. Automated Non-Coplanar VMAT Planning

Each case was also planned (either retrospectively or concurrently) using the automated non-coplanar VMAT technique (HyperArc) with the Eclipse treatment planning system, which automatically chooses from a selection of non-coplanar beam angles based on the target location (gantry 0–180°; couch 0, 45, 90, or 315°). The beam energy, dose rate, and MLC were the same as the conventional plans (6X-FFF, 1400 MU/min, and HD 120 MLC). By default, the isocenter for HyperArc plans is placed in the center of the PTV. However, due to the highly automated nature of HyperArc plan delivery, the permissible isocenter locations are limited to within a specific Patient Protection Zone to reduce the risk of collision. Since many of the head and neck targets in this study were considerably inferior and/or lateral to the intracranial target region for which HyperArc was initially designed, the isocenter automatically placed at the target center would be localized outside the patient-specific protection zone, and therefore, had to be manually adjusted for 13 out of 20 plans. An example of this isocenter shift is shown in Figure 1 (bottom).

All HyperArc plans utilized one half or full coplanar arc and two to three half non-coplanar arcs, which were automatically selected based on tumor location and patient anatomy, as illustrated in Figure 1. The collimator angle for each field was also automatically optimized based on the target location and beam angles to minimize leakage [25]. For HyperArc planning, the goal was to maintain clinically comparable OAR doses to the conventional plan while boosting the target dose as high as possible. Any maximum OAR doses less than 5 Gy or dose differences within approximately 3 Gy were typically considered clinically equivalent for the two plan types. The dose escalation was performed by re-optimizing the HyperArc plans until the target dose could not be increased any further without exceeding the OAR doses achieved in the corresponding conventional plan. As with the conventional plans, inhomogeneous target doses with hotspots in the center of the GTV were encouraged.

### 2.3. Data Analysis

The mean doses to the target volumes and maximum doses to the OARs were compared between the two plan types. The dose conformity index, R50%, and the gradient index (GI) were calculated as the ratio of the 50% isodose volume to the PTV volume and the 50% isodose volume to the 100% isodose volume, respectively. The gradient measure (GM), defined as the difference between the equivalent sphere radii of the 50 and 100% prescription isodose volumes, was also reported. The 100% isodose level was defined as the dose to 95% of the PTV for both plan types. The total MU for each plan type was also compared, and the plans for a subset of 10 patients were delivered on the treatment machine in quality assurance mode to estimate treatment delivery times.

Radiobiological modeling was also performed to predict any potential difference in patient outcomes between those treated with the conventional and HyperArc plans. The tumor control probability (TCP) and normal tissue complication probability (NTCP) values were calculated using an open-source MATLAB implementation of Niemierko’s effective uniform dose (EUD) model [26,27]. The NTCP for brainstem necrosis, cord myelitis necrosis, laryngeal edema, and reduced mandibular joint function were calculated for both plan types using the model parameters given in Table 2. Although there were several tumor types represented in this patient cohort, as shown in Table 1, the model parameters for squamous cell carcinoma were used for all TCP calculations for simplicity. Statistical significance for all parameters was determined using a paired sample *t*-test with a 2-tailed, 5% significance level.

## 3. Results

**Figure 2:**
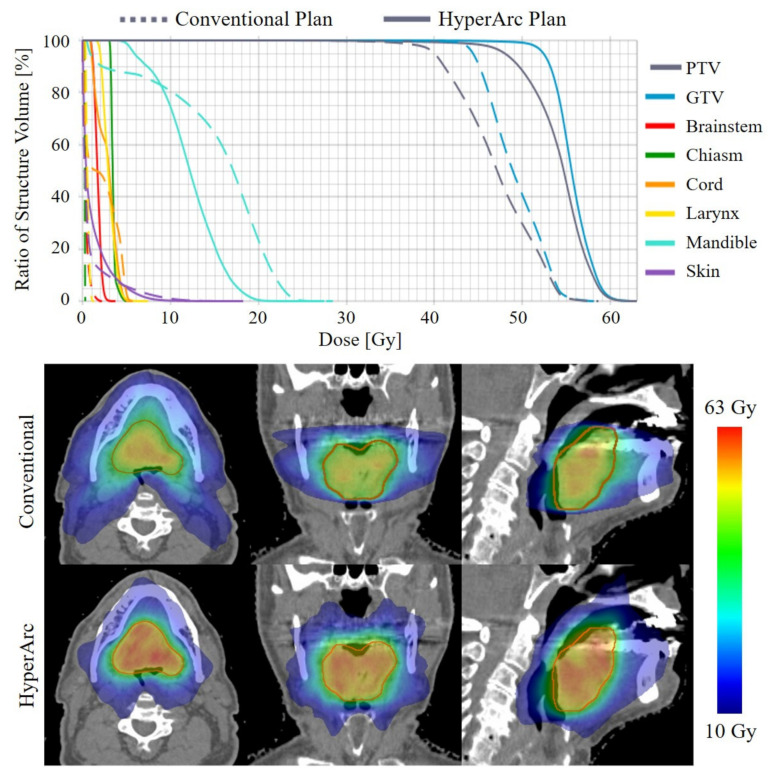
DVH (**top**) and dose distributions (**bottom**) for the conventional and HyperArc plans for one representative patient in the study (Patient 5).

**Figure 3:**
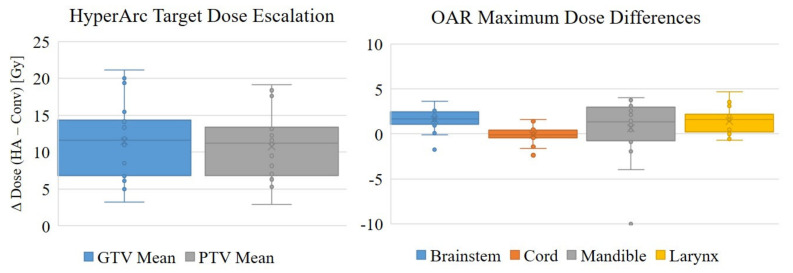
Target dose escalation and maximum OAR dose comparisons, plotted as the difference between the HyperArc (D_HA_) and conventional (D_Conv_) plan doses, in Gy.

Overall, the HyperArc treatment planning technique achieved conformal dose distributions with substantial dose escalation compared to the conventional plans and with comparable OAR doses. This is illustrated in the dose volume histograms (DVH) and dose distributions in Figure 2 for one representative patient in the study. The HyperArc plans achieved statistically significant increases in mean PTV and GTV doses by 10.8 ± 4.4 Gy (25 ± 11%) and 11.5 ± 5.1 Gy (26 ± 12%), respectively, compared to the conventional plans (Table 3). The escalation in the mean target dose for each HyperArc plan is plotted in Figure 3 (left). There were statistically significant improvements in GI and GM with HyperArc, and the R50% was slightly better with HyperArc, but not statistically significant.

Clinically equivalent OAR doses were maintained between the conventional and HyperArc plans. There were slight increases in dose to a few OARs with the HyperArc plans, and these increases were statistically significant for the brainstem, larynx, and optical structures. However, these were all well below the standard dose constraints, and the average differences in maximum dose between the two plan types for these organs were only 0.8 to 1.6 Gy.

The radiobiological modeling results, given in Table 4, suggest that treating with the escalated HyperArc plans rather than the conventional VMAT plans could significantly increase the probability of tumor control by up to 58% in some cases, with an average increase of 23 ± 21% (calculated for the PTV). There were no significant differences in NTCP for the brainstem, cord, mandible, or larynx, suggesting clinically equivalent OAR doses between the conventional and HyperArc plans.

## 4. Discussion

The HyperArc planning technique was originally developed as a mono-isocenter VMAT approach for stereotactic radiosurgery (SRS) of multiple brain metastases. SRS has become a widely used alternative to whole-brain radiation therapy for treating multiple metastases, and its efficacy has been demonstrated for treating as many as ten lesions per plan [31]. However, the added imaging time necessary for multiple-isocenter plans makes this approach impractical for patients with five or more metastases. HyperArc has enabled not only more efficient delivery of these intracranial treatments, but also more conformal dose distributions than conventional VMAT plans [21,22].

Compared with cranial stereotactic radiotherapy, non-coplanar planning is less used for HN reirradiation on C-arm radiotherapy systems due to concerns about patient collision and delivery efficiency. Robotic-based radiotherapy systems have the dosimetric advantage of non-coplanar delivery from wide beam angles with automated delivery; however, the delivery efficiency is relatively low due to its limited field sizes and complex delivery trajectories. Particle therapy, such as proton therapy, has intrinsic dosimetric advantages due to the Bragg peak, making it an ideal modality for HN reirradiation. However, there are considerable challenges for the wide adoption of this technique, such as cost, accessibility, and uncertainties associated with range and motion. This study demonstrated the dosimetric benefits of a widely available non-coplanar planning technique for HN reirradiation using existing C-arm linacs. Patient collision is prevented with automated beam selection, and high efficiency is achieved with automated delivery, making it a very competitive and cost-effective platform for HN reirradiation.

Due to the proximity of target volumes to numerous critical structures, head and neck radiation therapy cases are among the most difficult to plan and can result in significant toxicity. Therefore, the need for more conformal treatment techniques is even more significant for the HNC patient population. In this study, we demonstrated the feasibility of expanding the use of the HyperArc planning technique to a wide range of target locations in the head and neck. The potential for safe target dose escalation was also demonstrated, with significantly higher predicted tumor control probability for the HyperArc plans. Although there is always some uncertainty in absolute TCP and NTCP values due to the wide range of published model parameters, the relative probabilities between different plan types can be useful for evaluating potential differences in patient outcomes. Another limitation in this NTCP study is the lack of consideration for prior OAR doses. However, the prior dose distributions for many patients in this study were unavailable, which is part of the rationale for planning with such conservative dose constraints. Even for patients with known prior dose distributions, the cumulative NTCP estimation accuracy would have additional sources of uncertainty, such as image registration, dose summation, and recovery effect modeling.

An important consideration for this type of planning study is the influence of manual planning on the dosimetric results, including both inter- and intra-planner variability. As shown in a brain SRS planning study by Vergalasova et al. [24], large variations exist when using manual VMAT planning, while HyperArc may achieve more consistent plan quality because the software assists the user by automatically selecting the optimal isocenter, collimator rotation, and non-coplanar arc setup. While the focus of this study was to demonstrate the dosimetric benefits of HyperArc planning, we plan to perform a similar study as Vergalasova et al. to evaluate the advantage of using HyperArc automated planning to reduce variability in HN planning.

The highly inhomogeneous target dose distributions for these plans make it difficult to objectively compare dose conformity, which is why several different metrics were evaluated. The R50% was similar between the two plan types, but the gradient index, which is arguably less sensitive to the 100% and 50% isodose level definitions, was significantly lower for the HyperArc plans than the conventional plans. The gradient measure was also smaller for every HyperArc plan, with a statistically significant difference between both plan types.

While this study was focused on dose escalation and tumor control, a comparison of plan dosimetry quality with equivalent target coverage and prescription doses was reported in the recent study by Ho et al., which compared RapidArc and HyperArc planning techniques only for recurrent nasopharyngeal carcinoma patients [32]. Since there was no dose escalation in this study, one would expect substantial improvement in normal tissue sparing by the HyperArc plans. However, there were surprisingly no clinically significant reductions in OAR doses reported in this study, probably due to lack of experience implementing this new planning technique.

HyperArc beam angles are automatically selected according to the isocenter location from a fixed set of four couch angles, unlike the 4π radiotherapy technique where the ideal gantry and couch angles are selected using a complex optimization algorithm [8,17]. Nevertheless, the improvement in TCP is consistent with the previously reported 4π technique, which is not commercially available, thus difficult to deliver. In comparison, HyperArc is currently an accessible and efficient non-coplanar treatment option. The incorporation of couch angle optimization to further improve HyperArc planning is an area of interest for future studies.

Despite this limited use of optimization, the benefits of HyperArc over manual planning are not surprising. For manual planning, coplanar beams are usually preferred for the head and neck region because of the concern for patient collision. HyperArc always uses 2–3 non-coplanar beams, which naturally leads to dosimetric benefits over coplanar planning. Even if the planner is experienced in choosing non-coplanar beams in manual planning, the couch rotation is usually fairly small because it is difficult to predict patient collisions. The couch rotations can be large in HyperArc planning because patient collisions would be automatically detected, and this aggressive selection of non-coplanar beams in HyperArc further improves the plan dosimetry. The benefits of automated over manual non-coplanar beam selection have been well documented [8,11,18].

This study is limited to a dosimetric comparison between HyperArc and conventional head and neck plans, and a more thorough comparison of delivery aspects, such as treatment time, patient setup, and immobilization, will be detailed in a later report using data currently being gathered in a clinical trial at our institution. The patient setup uncertainty for HyperArc delivery is an area of concern—particularly for plans with multiple targets, since it has been shown that rotational setup errors can have significant dosimetric impacts for HyperArc plans with multiple brain metastases [33]. Since the HyperArc immobilization device was designed for intracranial treatment, it also may not be sufficient for the head and neck, especially for targets in the inferior neck. Data is currently being collected to evaluate intra-fraction motion and assess the need for better immobilization.

## 5. Conclusions

This study demonstrated an innovative approach for treating rHNC patients with the HyperArc treatment planning technique that can enable significant dose escalation for head and neck SBRT plans while achieving similar OAR doses. This will potentially improve local control and overall survival rates while limiting the risk of treatment-related toxicity. These positive results from a wide range of target locations suggest that HyperArc could also be beneficial for reducing toxicities in a wider population of head and neck cancer patients, including those receiving primary radiation therapy and non-escalated reirradiation.

## Figures and Tables

**Table 1:** Target and conventional plan characteristics for the 20 rHNC patients in the study.

Patient	Site	Histology *	PTV Volume (cm^3^)	Conventional Plan (arcs)	
				Coplanar	Non-Coplanar
1	Hypopharynx	SCC	49.5	2 half	0
2	Hypopharynx	SCC	34.2	4 partial	0
3	Submandibular gland	PTC	15.1	4 partial	0
4	Maxillary sinus	Mucosal melanoma	80.6	2 full	2 partial
5	Oral cavity	ACC	74.0	4 half	0
6	Larynx	SCC	52.2	4 half	0
7	Left neck	SCC	60.7	2 partial	0
8	Left neck	SCC	56.0	4 half	0
9	Right neck	SCC	77.1	2 partial	0
10	Cavernous sinus	ACC	9.2	2 partial	3 partial
11	Hypopharynx	SCC	7.3	2 partial	0
12	Right neck	Undifferentiated carcinoma	4.9	2 half	0
13	Right neck	SCC	38.8	2 half	0
14	Right neck	SCC	43.8	2 half	0
15	Supraclavicular nodes	SCC	13.8	0	2 half
16	Base of tongue	SCC	9.5	2 full	0
17	Temporalis	Salivary ductal carcinoma	44.5	2 half	0
18	Hypopharynx	SCC	5.4	0	2 half
19	Pharynx	Leiomyosarcoma	17.1	4 half	0
20	Tongue	SCC	9.5	2 full	0

* SCC, squamous cell carcinoma; ACC, adenoid cystic carcinoma; PTC, papillary thyroid carcinoma.

**Table 2:** Model parameters used for the EUD-based TCP and NTCP calculations.

Structure	Effect	α/β	*a*	γ_50_	*TCD_50_*/*TD_50_*
Target *	Long term tumor control	10.5	−8.0	3.2	66.8
Brainstem ^†^	Necrosis	3.0	7.0	3.0	65.0
Cord ^†^	Necrosis	3.0	7.4	4.0	66.5
Larynx ^†^	Edema	3.8	12.5	4.0	70.0
Mandible ^†^	Limited joint function	3.0	14.0	4.0	72.0

* Parameter *a* from Wu et al. [28], the rest from Stuschke et al. (*TCD_50_* averaged over five trials) [29]. ^†^ All parameters from Mesbahi et al. [30].

**Table 3:** Plan metrics and dosimetric statistics for the conventional and HyperArc plans (given as mean ± standard deviation).

	Conventional	HyperArc	Difference (HA–Conv)
Total MU	2237 ± 640	3183 ± 688	947 ± 873 *
Delivery Time (min, n = 10)	2.3 ± 0.6	4.3 ± 0.5	2.0 ± 1.0 *
R50%	4.1 ± 2.6	3.6 ± 1.7	−0.5 ± 1.6
GI	3.7 ± 1.4	3.0 ± 0.9	−0.7 ± 0.7 *
GM (mm)	9.8 ± 2.1	8.2 ± 1.7	−1.6 ± 1.3 *
	*Mean Dose (Gy)*		
PTV	43.2 ± 3.4	53.9 ± 5.9	10.8 ± 4.4 *
GTV	44.4 ± 3.7	55.9 ± 6.5	11.5 ± 5.1 *
	*Maximum Dose (Gy)*		
Brainstem	2.5 ± 5.8	4.1 ± 5.1	1.6 ± 1.2 *
Spinal Cord	5.6 ± 3.1	5.5 ± 3.3	−0.1 ± 1.0
Mandible	10.4 ± 11.7	11.0 ± 8.5	0.6 ± 3.3
Larynx	14.9 ± 18.9	16.4 ± 19.1	1.5 ± 1.5 *
Skin	25.5 ± 11.4	25.8 ± 11.9	0.3 ± 2.7
Right Optic Nerve	1.3 ± 3.2	2.7 ± 3.1	1.4 ± 1.8 *
Left Optic Nerve	1.5 ± 4.2	2.3 ± 3.2	0.8 ± 1.8 *
Chiasm	1.1 ± 2.7	2.3 ± 3.0	1.2 ± 1.3 *

* Statistically significant difference (paired, 2-tailed *t*-test, *p* < 0.05).

**Table 4:** The calculated tumor control probability (TCP) and normal tissue complication probability (NTCP) for the conventional and HyperArc plans (given as mean ± standard deviation).

	TCP (%)		NTCP (%)			
	GTV	PTV	Brainstem	Cord	Mandible	Larynx
Conventional	43.8 ± 31.1	28.6 ± 23.5	0	0	4.4 ± 19.5	15.6 ± 31.2
HyperArc	61.4 ± 42.5	51.5 ± 40.8	0	0	0.1 ± 0.4	17.5 ± 33.0
Difference (HA–Conv)	17.6 ± 15.0 *	22.9 ± 20.6 *	0	0	−4.3 ± 19.1	1.9 ± 5.9

* Statistically significant difference (paired, 2-tailed *t*-test, *p* < 0.01).

## Data Availability

The data presented in this study are available in this article.
